# Metastatic Recurrence of Breast Cancer by Stage and Molecular Profile: A Population‐Based Study Among Italian Women

**DOI:** 10.1002/cam4.71492

**Published:** 2026-01-04

**Authors:** Fabiola Giudici, Sara De Vidi, Stefano Guzzinati, Federica Toffolutti, Silvia Francisci, Angela B Mariotto, Alessandra Ravaioli, Laura Botta, Roberta De Angelis, Ettore Bidoli, Fabio Falcini, Antonella Puppo, Lucia Mangone, Maria Michiara, Mario Fusco, Anna Clara Fanetti, Alessandra Andreotti, Riccardo Capocaccia, Diego Serraino, Luigino Dal Maso, AIRTUM working group, Angela De Paoli, Elena Demuru, Daniela Pierannunzio, Silvia Rossi, Andrea Tavilla, Gemma Gatta, Paolo Contiero, Giovanna Tagliabue, Martina Taborelli, Claudia Casella, Isabella Bisceglia, Ilaria Fontanili, Maria Francesca Vitale, Paola Giumelli, Maddalena Baracco, Adele Caldarella, Lucia Bisceglia, Enrica Migliore, Maria Letizia Gambino, Margherita Ferrante, Fabrizio Stracci, Cinzia Gasparotti, Giuliano Carrozzi, Rossella Cavallo, Walter Mazzucco, Paola Ballotari, Giuseppe Sampietro, William Mantovani, Lorenza Boschetti, Giuseppe Cascone, Michael Mian, Maria Teresa Pesce, Daniela Piras, Rocco Galasso, Francesca Bella, Pietro Seghini, Pasquala Pinna

**Affiliations:** ^1^ Cancer Epidemiology Unit Centro di Riferimento Oncologico di Aviano (CRO) IRCCS Aviano Italy; ^2^ Epidemiological Department Azienda Zero Padua Italy; ^3^ National Centre for Disease Prevention and Health Promotion National Institute of Health Rome Italy; ^4^ Surveillance Research Program, Division of Cancer Control and Population Sciences National Cancer Institute Bethesda Maryland USA; ^5^ Emilia‐Romagna Cancer Registry, Romagna Unit IRCCS Istituto Romagnolo per lo Studio dei Tumori (IRST) “Dino Amadori” Forlì Italy; ^6^ Evaluative Epidemiology Unit, Department of Research Fondazione IRCCS Istituto Nazionale dei Tumori di Milano Milan Italy; ^7^ Department of Oncology and Molecular Medicine National Institute of Health Rome Italy; ^8^ Liguria Cancer Registry IRCCS Ospedale Policlinico San Martino Genova Italy; ^9^ Emilia‐Romagna Cancer Registry, Reggio Emilia Unit, Epidemiology Unit Azienda Unità Sanitaria Locale—IRCCS di Reggio Emilia Reggio Emilia Italy; ^10^ Emilia‐Romagna Cancer Registry, Parma Unit, Medical Oncology Unit University Hospital of Parma Parma Italy; ^11^ UOSD Registro Tumori ASL Napoli 3 Sud Napoli Italy; ^12^ Registro Tumori ATS della Montagna, Agenzia di Tutela della Salute della Montagna Sondrio Italy; ^13^ Editorial Board Epidemiologia e Prevenzione Milan Italy

**Keywords:** breast cancer, metastatic recurrence, mixed cure model, relative survival

## Abstract

**Background:**

This study aims to estimate the long‐term risk of metastatic recurrence (MR) among Italian women with breast cancer (BC) by period, age, stage, and surrogate molecular profile.

**Methods:**

Data on 59,968 women below age 75 years diagnosed in 1997–2017 with stage I‐III BC from 7 population‐based Italian cancer registries were analyzed. We used a novel modeling method, based on an illness–death process coupled with a mixture cure model, to estimate relative survival and MR risks up to 15 years after BC diagnosis according to calendar period, age, stage, and profile.

**Results:**

The risk of MR for the entire cohort at 15 years decreased from 20.6% in 1997–2006 to 12.3% in 2007–2017, when MR risk within 15 years was 3.0% for stage I, 16.0% for stage II, and 42.7% for stage III. The conditional risk of MR decreased with time since diagnosis, with stage I–III triple‐negative BC having a higher risk of developing MR in the first 5 years regardless of age (16.0% at age 15–54 years and 18.3% at 55–74 years), but < 1% once they survived for 5 years without recurrence. In contrast, hormone receptor‐positive BC had a lower but persisting risk of MR of about 6% for both age groups in the first 10 years, halving to about 3% in the following 5 years after diagnosis.

**Conclusions:**

This study provides a population‐based estimate of the long‐term risk of MR for women with BC by major prognostic factors. These findings may help in tailoring follow‐up strategies through informative risk stratification.

**Abbreviations**
BC: breast cancer
CR: cancer registries
ER: estrogen receptor
HER2: human epidermal growth factor receptor 2
HR: hormonal receptor
ICD‐10: International Classification of Disease 10th version
MR: metastatic recurrence
PR: progesterone receptor
TN: triple negative

## 1 Introduction

As of January 2020, it was estimated that more than 5 million women lived in Europe after a breast cancer (BC) diagnosis [1], and there will be 1 million in Italy in 2030 (3.6% of all Italian women) [2]. The number of BC survivors continues to increase by 2.2% per year in Italy [2], as elsewhere [1, 3], and most of them will remain in remission and will experience the same life expectancy as the general population [4, 5]. However, while 5% are diagnosed with de‐novo stage IV disease, studies have shown that about a quarter of patients with early‐stage disease will eventually progress to metastatic breast cancer [6, 7].

Advances in the treatment of metastatic BC have been slow, with median survival remaining stable at 2–3 years [8] and, once metastatic, BC is traditionally considered an incurable condition. This assumption has remained unquestioned for a long time; nevertheless, it is now challenging due to the advances in new targeted therapies, thanks to which women with metastatic BC are surviving for several years after treatment [9]. This trend was also confirmed by the projection of recently published American studies, which reported that one‐third of women are expected to live 5 years or more after metastatic recurrence (MR) [10, 11].

Although women with metastatic BC are living longer [12], it is important to emphasize that disease progression heavily affects the quality of life in terms of physical and mental health [13, 14], and the fear of recurrent cancer is very common in BC survivors [15]. Consequently, providing population‐based information on MR until 15 years after BC can supply a more comprehensive characterization of the disease course and help clinicians delineate women who may be candidates for more or less intense or prolonged follow‐up. Moreover, since advanced BC had a significant impact not only on physical health but also on psychological well‐being, more accurate estimates of the risk of MR could support the work of advocacy groups, psycho‐oncologists, nurses, and oncologists who aimed to detect the specific needs and concerns of women with metastatic BC and improve the quality of life for all patients with BC [16, 17, 18].

Estimating the percentage of women diagnosed with early‐stage BC who later have had MR is, however, challenging. Currently, population‐based registration of intermediate outcomes is extremely resource‐consuming and not performed by most registries [19, 20]. To overcome the lack of these data, a method has been recently developed to estimate the MR risk, using stage‐specific cancer registry survival data with published information on survival from recurrence [10]. This modeling approach also estimates the conditional MR risk, i.e., the probability of progressing to MR in a time interval given being alive and recurrence‐free at the beginning of the interval. Taking into account that survival and risk of recurrence are time‐dependent, conditional survival estimates are needed to inform women who are alive several years after diagnosis.

This population‐based study aimed to investigate the MR risk up to 15 years after primary diagnosis for Italian women with early‐stage BC, applying the model proposed by Mariotto et al. [10]. The MR risk was estimated overall and according to the period of diagnosis, age, stage, and surrogate molecular profile.

## 2 Materials and Methods

### 2.1 Study Population

The study included a population‐based (unselected) cohort from seven Italian cancer registries (covering 9% of the Italian population). These registries provided stage information for at least two‐thirds of the malignant BC patients (International Classification of Diseases, tenth edition [ICD‐10] C50) in each calendar year of registration and for at least 15 consecutive years in the period 1997–2017. They also provide women's vital status ascertainment at least 1 year after the last incidence date and a complete follow‐up until December 31, 2018 [21]. The 7 registries have a duration of registration ranging from 19 to 21 years and include 90,280 incident cases of malignant BC (Table S1).

Women older than 75 years (*n* = 22,757, 25.2%) and with missing stages (*n* = 7,555, 11.2%) were excluded. The final cohort included 59,968 women (Table 1). The TNM (6th and 7th editions) was applied to define the stage at diagnosis. According to the Italian and international rules, TNM staging was based on histopathological examination (pTNM) if available, on the clinical examinations which led to diagnosis otherwise. For four cancer registries (Table S2), hormonal receptors (HR) (i.e., estrogens [ER]/progesterone receptors [PR]) and human epidermal growth factor receptor 2 (HER2) were available since 2003 for at least two‐thirds (67%) of cases allowing for the classification of BCs into three BC subtypes [22, 23]: HR+/HER2− (ER+ or PR+ and HER2−), HER2+ (HER2+ and any ER/PR status), and Triple Negative (TN, ER−/PR− and HER2‐) (Table S3). For this cohort, the MR risk, according to age, stage and surrogate molecular profile, was estimated for the period 2003–2012 with a follow‐up on 31st of December 2018, including 18,573 women.

**TABLE 1:** Breast cancer cases in Italian cancer registries contributing to the present study^a^ by stage and age at diagnosis.

Stage	Age groups (years)									
	15–74		15–44		45–54		55–64		65–74	
	*N*	%	*N*	%	*N*	%	*N*	%	*N*	%
I	30,618	51.1%	3,528	42.1%	8,087	51.3%	9,300	53.9%	9,703	52.3%
II	20,415	34.0%	3,394	40.5%	5,524	35.1%	5,516	31.9%	5,981	32.2%
III	6,907	11.5%	1,187	14.2%	1,726	11.0%	1,863	10.8%	2,131	11.5%
IV	2,028	3.4%	271	3.2%	420	2.7%	589	3.4%	748	4.0%
All stages	59,968	100%	8,380	100%	15,757	100%	17,268	100%	18,563	100%

^a^ Breast cancer cases aged 15–74, diagnosed in 1997–2017 and followed up until 2018.

### 2.2 Overview of the Statistical Method

The method developed to estimate the risk of MR for women with breast cancer in the United States [10] was applied to the Italian population. The term “metastatic recurrence” refers to both the presence of distant metastasis following an early‐stage cancer with a disease‐free period and the progression of an early‐stage cancer to a metastatic disease without any disease‐free period. In this analysis, local or regional recurrences after an early‐stage diagnosis are not included in the estimation of “metastatic recurrence”, as well as the occurrence of metastasis at BC diagnosis. This method is based on the diagnosis–metastasis–death pathway (Figure S1). We used a mixture cure survival model to estimate the proportions of patients that at diagnosis are cured and not at risk of dying of their cancer (c) and of patients who are not “cured” (1‐c), who eventually will die of cancer and for this last group, with their survival time (T*). The method assumes that patients in the noncured fraction progress through recurrence or progression with metastatic disease before dying of cancer [24]. In the absence of deaths from other causes, the survival time for the non‐cured (T*) can be defined as the sum of the times from diagnosis to metastasis (T_1_, i.e., the recurrence free time), the quantity we aimed to estimate, and from metastasis to cancer death assumed to be known (T_2_) (T* = T_1_ + T_2_, Figure S1). Survival time from recurrence to death (T_2_) is estimated by combining cancer registry relative survival from de novo stage IV and an estimate of the higher or smaller risk of cancer death mortality after recurrence with metastatic disease (r, mortality hazard ratio). By knowing the survival distributions of T * and T_2_, we can then extract the recurrence‐free survival (T_1_).

In brief, to estimate the time to metastasis for women in the “non‐cured” fraction, two pieces of information are combined: (i) relative survival from the population‐based registries and (ii) mortality hazard ratio of metachronous metastatic recurrence with respect to de novo metastatic diagnosis from published studies.

Assuming independence, the survival time for those not cured (T*) is the sum of the time from diagnosis to MR (T_1_) and the time from MR to cancer death (T_2_) (Figure S1). Since the risk of death is higher for cases developing MR than for those with metastases at diagnosis, survival time from MR to death (T_2_) is estimated by adjusting the survival of stage IV BC for a factor *r* (i.e., MR survival = (Stage IV BC survival)^*r*^), which represents a cause‐specific mortality rate ratio, i.e., a mortality hazard ratio and can be retrieved from previously published studies [25]. By knowing the distributions of survival times T* and T_2_, we can extract the recurrence‐free survival (S_1_) using two “subtraction” methods that provide comparable results: analytical and numerical. In this paper, we report the analytical results. Once we estimate the distribution of T_1_ and its survival, the probability of being recurrence‐free is estimated as c + (1‐c) S_1_(t). Further technical details have been described in Supporting Information Methodological Material.

The application of the method consisted of three steps, summarized in Figure S2. First, we estimated relative survival for patients diagnosed with BC by period of diagnosis (1997–2006, 2007–2017), age group (15–44, 45–54, 55–64, 65–74), and stage (I, II, III, IV), using the actuarial method and Ederer II approach, as implemented by SEER*Stat software [26]. We excluded patients identified by death certificate or autopsy, and those with zero months of survival. Second, we fitted a mixture cure survival model to relative survival using the CanSurv Version 1.4 software [27, 28] to estimate the cure fraction c and the survival for the uncured patients, by stage (I, II, and III), age at diagnosis, and period of diagnosis. The age groups and period of diagnosis were entered as categorical covariates in the model, while the stage at diagnosis was used as a stratification variable. The final step consists of uploading the relative survival data together with the output from CanSurv into the RecurRisk web tool [29], where it is required to specify the adjustment factor *r*. de Maar's [25] (Figure 2; Table 2) results showed a hazard ratio (HR) of mortality of 0.75 in BC cases metastatic at diagnosis, compared to those with subsequent metastasis. Based on this estimate, we used the adjustment *r* of 1.33 (HR = 1/0.75 = 1.33), which should be interpreted as indicating a 33% higher probability of death from breast cancer after metastatic recurrence than after a de novo metastatic breast cancer. Given the availability of more than 10 years of follow‐up data, we reported the risk of metastatic recurrence at 5, 10, and 15 years after diagnosis. To evaluate the impact of the adjustment factor r, the risk of metastatic recurrence was provided using a range of r values from 1.00 to 1.70.

**TABLE 2:** Conditional probabilities of metastatic recurrence risk for Italian breast cancer patients diagnosed in 1997–2017 by stage, age group, and period.

Stage	Period of diagnosis	Years since diagnosis	Age groups (years)				
			15–44	45–54	55–64	65–74	15–74
I	1997–2006	0 to 5	3.8%	1.9%	2.5%	1.6%	2.1%
		5 to 10	4.0%	2.1%	2.9%	1.9%	2.8%
		10 to 15	3.3%	1.8%	2.5%	1.6%	2.4%
	2007–2017	0 to 5	2.7%	1.4%	1.8%	1.1%	1.0%
		5 to 10	2.7%	1.4%	1.9%	1.2%	1.2%
		10 to 15	2.1%	1.1%	1.6%	1.0%	0.9%
II	1997–2006	0 to 5	16.6%	10.5%	13.4%	14.7%	13.5%
		5 to 10	11.4%	7.9%	10.3%	12.2%	10.3%
		10 to 15	6.3%	4.7%	6.3%	7.8%	6.1%
	2007–2017	0 to 5	10.5%	6.8%	8.5%	9.2%	8.4%
		5 to 10	6.2%	4.1%	5.6%	6.8%	5.5%
		10 to 15	3.0%	2.1%	3.0%	4.0%	2.9%
III	1997–2006	0 to 5	37.8%	32.8%	37.5%	42.5%	38.2%
		5 to 10	16.5%	17.4%	21.3%	28.6%	21.4%
		10 to 15	4.9%	6.8%	8.8%	13.8%	8.7%
	2007–2017	0 to 5	30.2%	27.7%	31.4%	36.1%	31.0%
		5 to 10	10.0%	10.2%	13.6%	20.2%	13.2%
		10 to 15	2.0%	2.7%	4.3%	8.3%	4.2%
I–III	1997–2006	0 to 5	14.6%	8.8%	10.5%	11.4%	11.0%
		5 to 10	8.7%	5.8%	7.1%	8.2%	7.3%
		10 to 15	4.2%	3.1%	3.7%	4.6%	3.7%
	2007–2017	0 to 5	9.9%	6.1%	7.1%	7.4%	7.0%
		5 to 10	5.1%	3.3%	4.1%	4.9%	4.0%
		10 to 15	2.1%	1.5%	1.9%	2.5%	1.8%

For patients diagnosed with BC in the period 2003–2012, we applied the same methodology to estimate the risk of MR also by surrogate molecular profile (HR+/HER2‐, HER2+, TN), as well as by stage (I, II, III) and age group (15–54, 55–74). The choice of the period of diagnosis and only two age groups ensures that no methodological problems arise in the estimates (see Supporting Information Methodological Material for further details).

In estimations of the MR risk by stage and surrogate molecular profile, Weibull and log‐logistic cure models were explored. The log‐logistic model has a better fit in estimating survival in general. However, the Weibull model was used for stage estimates as the log‐logistic model did not converge in some strata.

## 3 Results

The characteristics of the study cohort based on BC stage and age at diagnosis are shown in Table 1. Of the 59,968 female BC women included in the analysis, 96.6% were diagnosed with stage I–III, whereas 2028 women (3.4%) had stage IV. Women diagnosed with stage IV BC were not included in the study cohort; however, their survival data were used to estimate the survival following MR, as specified in the methods section. Among patients aged less than 44 years at diagnosis, 42.1% had stage I disease as compared to 53.9% for those diagnosed between 55 and 64 years and 51.1% for the entire cohort. Moreover, younger women had higher percentages of stage III disease than other age groups (14% vs. about 11%, respectively) (Table 1). For 18,573 women, data on HR and HER2 status were available (Table S3). Among them, 14,138 (76.1%) had HR+/HER2‐, 2917 (15.7%) HER2+ and 1518 (8.2%) TN disease. The distribution of surrogate molecular profiles varied according to the stage: among stage I the most represented profile was HR+/HER2‐ (7,804 out of 9,603, 81.3%) compared to HER2+ (12.2%) and TN (6.5%) subtypes while, considering women with stage III there was a lower proportion of HR+/HER2‐ tumors (68.2%) in favor of more aggressive profiles (HER2+ 22.6% and TN 9.2%) (Table S3).

**FIGURE 1:**
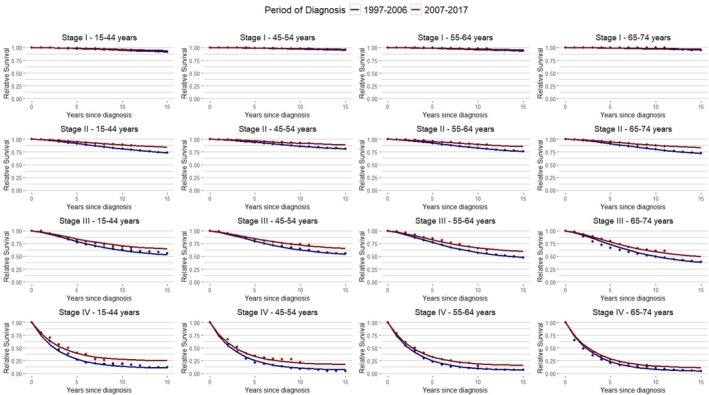
Relative survival (●) and corresponding model‐based estimates (lines) for Italian breast cancer patients diagnosed in 1997–2017 by stage, age group, and period.

In Figure 1, observed and model‐based relative survival is reported up to 15 years after diagnosis by age and stage. The BC relative survival is higher for women diagnosed in the most recent period (2007–2017), particularly for stage II‐IV BC, while women with stage I BC had relative survival > 90% at 15 years since diagnosis, regardless of age (Figure 1; Table S4).

Table S5 and Figure 1 show a substantial decrease in the overall 15‐year risk of MR between the two diagnostic periods from 20.6% for BC cases diagnosed in 1997–2006 to 12.3% in 2007–2017. Moreover, the decrease is consistent at all ages and stages. The largest decline in MR between the two periods was observed for stage III BC (15‐year risk difference of 12.9%), while for women with stage I BC, the decrease is slight (15‐year risk difference of 4.2%).

**FIGURE 2:**
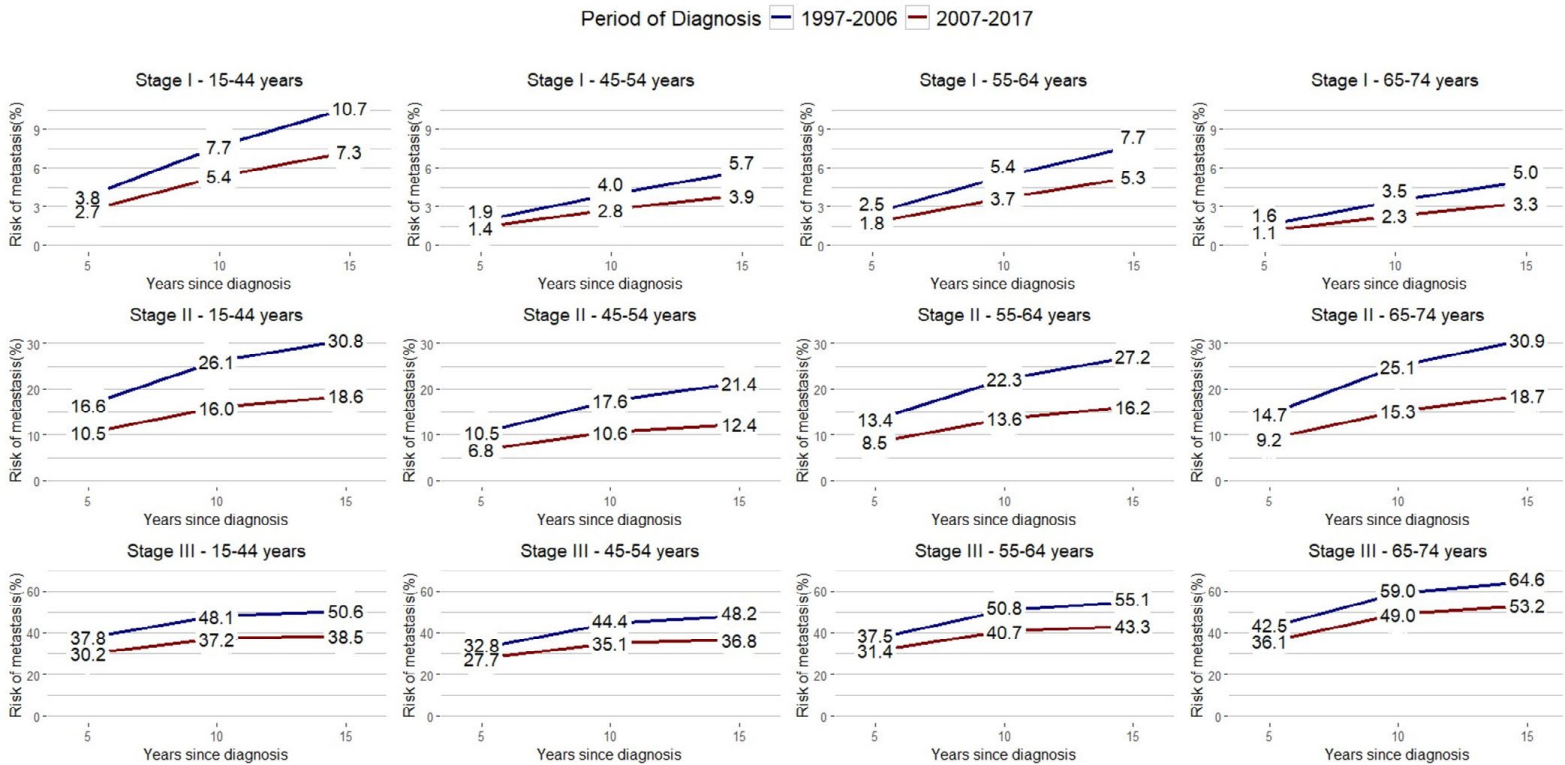
Risk of metastatic recurrence for Italian breast cancer patients diagnosed in 1997–2017 by stage, age group, and period.

Focusing on the recent period, 2007–2017, MR risk within 15 years was 3.0% for stage I, 16.0% for stage II, and 42.7% for stage III BC. The risk of MR varied by age and stage (Table S5; Figure 2). In particular, the women aged 15–44 years diagnosed with stage I BC in 2007–2017 had a higher risk of MR than other age groups, and the MR risk increased steadily with length of follow‐up, from 2.7% at 5 years to 7.3% at 15 years. In the oldest age group (65–74 years) with stage I BC in 2007–2017, MRs are lower both in the first 5 years after diagnosis (1.1%) and at 15 years (3.3%). This trend changes completely when considering women with stage III, in which we observed a marked increase in risk in older women (from 36.1% at 5 years to 53.2% at 15 years) compared to women in the 15–44 age group, whose risk in the first 5 years is 30.2% and reaches 38.5% at 15 years.

Table 2 shows the conditional probabilities of MR by stage, age group, and period of diagnosis, i.e., the probabilities of developing MR in the next 5 years, given that patients were alive and recurrence‐free at 0, 5, and 10 years since diagnosis. These conditional probabilities consistently decreased with time since diagnosis. Considering the diagnosis period 2007–2017, conditional risks decreased with time and became 1.8% between 10 and 15 years after diagnosis, although significant differences are found according to stage at diagnosis. The conditional probabilities of MR decreased remarkably with survival time for stages II and III. In particular, women with stage III BC had a higher MR risk (31.0%) in the first 5 years, which became 4.2% between 10 and 15 years since diagnosis. This trend is not experienced among women diagnosed with stage I BC, where the conditional risks of MR, even if very small, mostly remain similar with survival time, from 1.0% until 5 years to 0.9% between 10 and 15 years after diagnosis.

**FIGURE 3:**
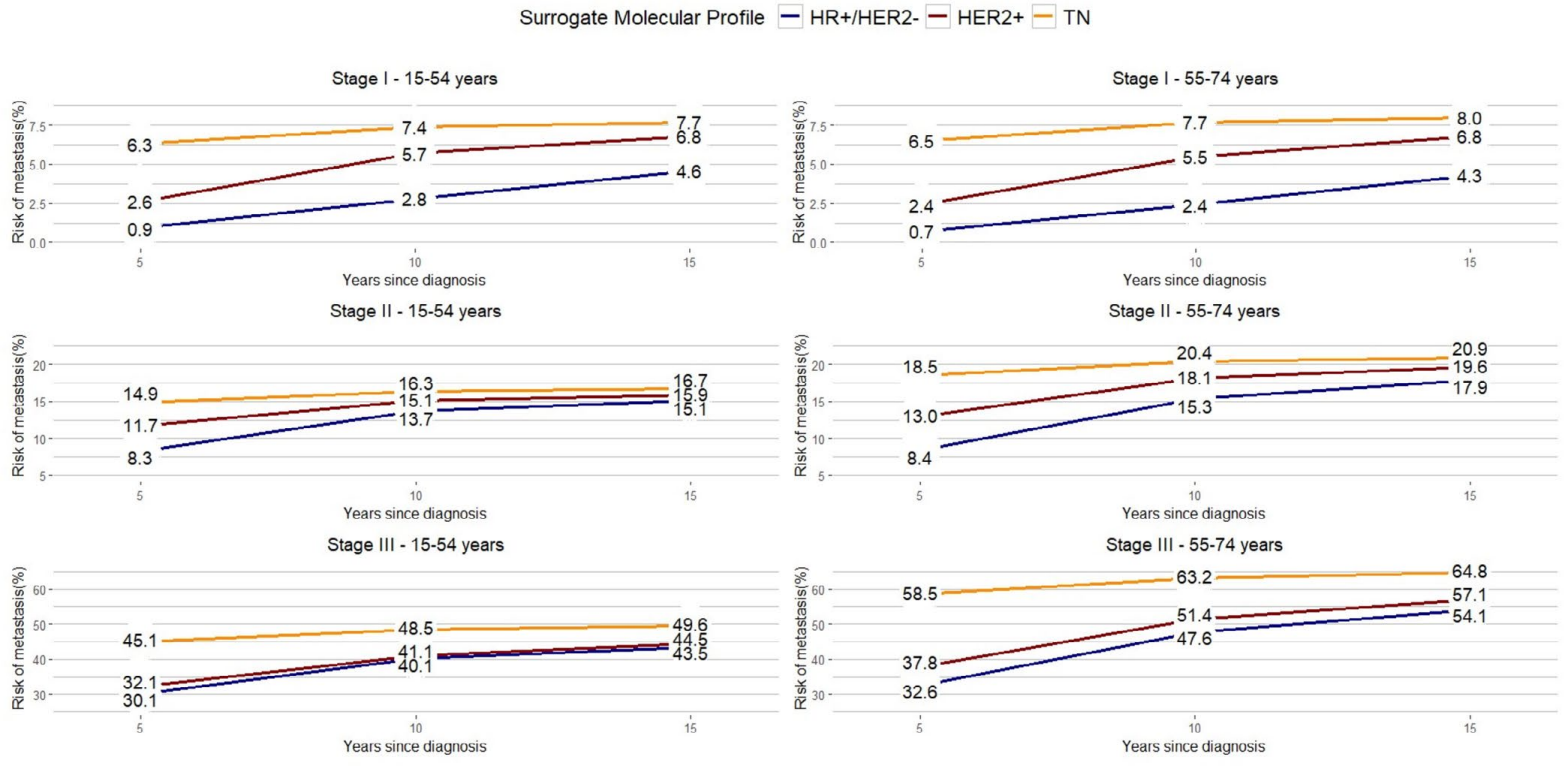
Risk of metastatic recurrence for Italian breast cancer patients diagnosed in 2003–2012 by stage, age group, and surrogate molecular profile.

**FIGURE 4:**
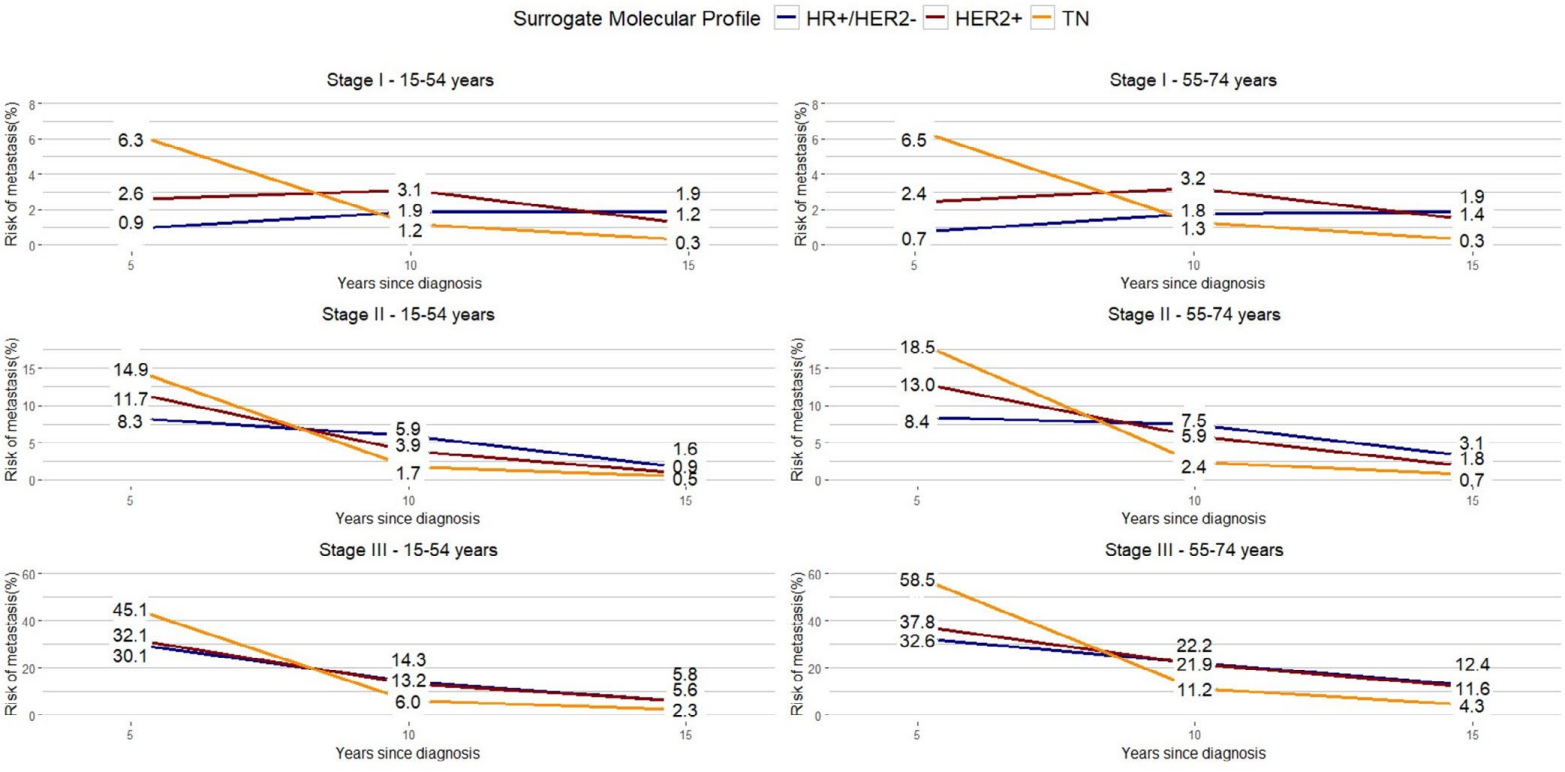
Conditional probabilities of metastatic recurrence risk for Italian breast cancer patients diagnosed in 2003–2012 by stage, age group, and surrogate molecular profile.

The cumulative risks of MR by stage, age group, and surrogate molecular profile are shown in Figure 3 and the corresponding conditional probabilities in Figure 4. Risks of MR within 5, 10, or 15 years were lower in women with HR+ tumors than the other two surrogate molecular profiles for all stages of the disease (Figure 3; Table S6). Women with TN BC (stages I‐III) had a higher risk of developing MR in the first 5 years regardless of age (16.0% at age 15–54 years and 18.3% at age 55–74 years), but once they survived for 5 years or 10 years without disease, this conditional risk became < 1% in 5 years (Figure 4; Table S7). A similar risk pattern was observed for HER2+ BC: higher risk in the early 5 years after diagnosis (about 12% for both age groups) with a decline to 1.3% and 2.1% for women aged 15–54 and 55–74 years, respectively. In contrast, HR+ BC showed a risk of MR persisting for a long time, with a rather stable trend in the first 10 years (about 6.0%) and declining slightly but still present at 15 years after diagnosis (2.8% and 3.8% in women aged under 54 and over 54, respectively).

A sensitive analysis has been performed (Tables S8–S10) to evaluate the impact of the choice of the adjustment factor r on MR risks at 5, 10 and 15 years for several strata of age, stage, and molecular profile. We found that the estimated risk of MR was robust to r. In particular, negligible differences for different r values (1.00, 1.50, 1.70) were observed as the years since diagnosis increased in all groups and very small differences for triple negative tumors.

## 4 Discussion

This study provides the first population‐based estimates of long‐term MR risk for Italian women with BC, and risk variations by period of diagnosis, age, stage, and surrogate molecular profile.

The decrease in MR risk is relevant between the two decades examined (20.6% in 1997–2006, 12.3% in 2007–2017), in particular for stage III BC.

The changes in treatment undoubtedly account for some of the decreases in breast cancer MR risk observed over the study period (1997–2017), in particular, the use of trastuzumab in the adjuvant setting for HER2+ women [30]. Other factors, such as increased BC awareness, screening, and more sensitive breast imaging techniques, are also likely to have contributed to reducing MR risk [31].

Our results are consistent with the study of Mariotto et al. [10], whose estimates of MR risk were obtained with the same modeling approach. The authors reported that 17.6% of women diagnosed with breast cancer in 2000–2013 (SEER data) will progress to metastatic BC within 15 years of diagnosis (20.6% in our study for the 1997–2006 period). Moreover, similar trends were found in terms of MR risk pattern of period of diagnosis (a decline in the most recent periods), stage (higher risk in advanced stage), and of HR+ and HR‐ tumors, even if not directly comparable because of the unavailability of the HER2+ variable in that study. This consistency is quite reassuring concerning the reproducibility of the method, given that it has been applied to very different populations and with different health systems.

Among population‐based cohorts that routinely collected MR information, few studies estimated long‐term recurrences: a study from Australia [32] used cancer registry data to investigate the long‐term risk of metastases in women diagnosed with non‐metastatic breast cancer and found that 22.2% of women had a metastasis within 14 years of follow‐up, similar to the 19.1% estimate observed in the current study. A Switzerland registry‐based [33] study estimated the MR risk according to surrogate subtype, reporting a 10‐year cumulative incidence for Luminal A tumors of 10% and for TN cancers of 25%, comparable to our estimates (9.6% and 23.5%, respectively).

Results from the Netherlands Cancer Registry, which analyzed the 10‐year conditional risk of MR in the prognostic subgroups [34], were consistent with our study. Patients with TN and HER2+ disease showed a higher risk of MR (about 18%) than Luminal A (7%), and their risk of recurrence declined over time, becoming as low as 0% for HER2+ and TN cancers, and 1.8% for Luminal A disease in the 10th year. The decline of MR risk over time was also documented by a more recent population‐based Australian study [35], when new adjuvant therapies became available in Australia, as elsewhere.

With regard to the difference in MR risk across age and stage groups, our findings were consistent with previous studies, which suggested that the higher MR risk in stage III BC is due to older patients less frequently receiving chemotherapy. Especially in TN and HER2+ BC, chemotherapy and anti‐HER treatments are less frequently used in older patients, mostly due to comorbidity, with higher MR risk [36]. In contrast, less aggressive treatment in older women with Stage I tumors did not appear to affect MR risk. In our study, higher late MR risk emerged for women aged 15–44 years with stage I BC, as compared to older age groups. Several population‐based studies have highlighted young age as an independent risk factor associated with unfavorable outcomes specific to breast cancer, concluding that younger age at diagnosis had a more frequent association with clinicopathologic features and gene expression for worse prognosis. In particular, Nixon et al. [37] showed that younger breast cancer patients at the early stage of disease had a worse prognosis than older ones and that this difference is only partially explained by a higher frequency of adverse pathologic factors seen in younger patients. The findings reported by a recently published study [38] suggested that age is an independent risk factor associated with late distant recurrence in young patients and stressed the issue of non‐adherence to treatment in the younger patients who were more likely to face significant fertility concerns and attempts to conceive, which could have influenced their adherence to therapy. Additionally, the adverse effects of endocrine therapy might have contributed to decreased adherence among younger patients [39]. Our results were in line with this literature, even if, unfortunately, the current study is not able to explore the adherence to treatments by age, since these variables are not currently collected by cancer registries.

It should be noted that in recent years, with rapid advances in computer technology and artificial intelligence [40], machine learning algorithms have been applied to estimate the risk of metastasis in cancer patients [41, 42]. Although these models have demonstrated excellent performance in external validation cohorts, the data originated from a single institution, which may limit the generalizability of the model. Furthermore, from a patient‐centered perspective, artificial intelligence algorithms will be able to support therapeutic choices that should be made by the patient and clinicians in collaboration with each other. They will also be able to provide tools, including the risk of recurrence, that contribute to a more comprehensive view of quality of life and its trends [18].

### 4.1 Limitations and Strengths

Our estimates of MR risk are based on a modeling method that relies on key assumptions which are reported and discussed in the Supporting Information Methodological Material. These assumptions must be verified to obtain reliable estimates. Moreover, the estimates are related to the choice of the mortality hazard ratio (*r* = 1.33), which is used to obtain the survival from recurrence, adjusting the survival from de novo metastatic cancer. This computation was justified by several studies which have evaluated the difference in prognosis between patients with stage IV at diagnosis and patients with a MR during the follow‐up, showing a worse prognosis in the second group (*r* > 1). Sensitivity analysis provided that our results are robust across a wide range of plausible adjustments.

Notably, MR risk was calculated after excluding the probability of dying from other causes (i.e., competing risks), an assumption that may result in an overestimated MR, especially in the older age group [43]. For the model including also stratification by surrogate molecular profile, only observations from 2003 to 2012 and from two age groups were used. These restrictions satisfy the key assumption of convolution of times T_1_ and T_2_ (Supporting Information Methodological Material). Even if our estimates are quite consistent with those previously reported, the method was not validated using external recurrence data collected from clinical charts (generally considered to be the gold standard), as no such data were available. However, this same model has been validated [44] for MR risk estimates 5 years after diagnosis, showing very similar results between intensive medical record review and modeling approach.

Some impact of the use of different mixture cure models (Weibull, log‐logistic, others) on the estimate of survival and MR is possible. The use of different models may provide estimates that diverge greatly when projected beyond the end of follow‐up, as is often done for mixed cure models that asymptotically estimate the probability of cure (i.e., of dying from causes other than cancer) [45]. However, as shown in Figure S3, the estimates of survival obtained for up to 15 years after diagnosis using the Weibull and log‐logistic models (when based on 15 years of observations) are almost identically distributed.

Finally, we excluded women aged more than 75 years and with missing stage of disease. In particular, older women constitute a particular subgroup in terms of comorbidity and treatments, and it would certainly be appropriate to conduct a specific study, perhaps limiting it to a shorter follow‐up (5 or 10 years). As regards stage, in our study, patients with missing stages have relative survival that is “intermediate” compared to the others. It is therefore plausible to assume that the missing stages of the disease are mainly due to the incompleteness of the information flows adopted by the registries and did not determine a relevant bias.

Nevertheless, our study has several strengths. Firstly, the use of data from large population‐based Italian cancer registries resulted in high completeness and representativeness, which allowed the generalisability of the estimates. Secondly, we provide conditional probabilities of MR according to the most important prognostic factors rarely reported, especially until 15 years after diagnosis.

Conditional survival analyses are methods to estimate the survival probability for patients who have survived for a certain period after initial diagnosis. Conditional survival estimates are therefore dynamic and more accurately reflect the current prognosis after initial management, therefore providing important information during follow‐up for both patients and physicians. Despite these advantages making it a state‐of‐the‐art method, conditional estimates have only recently begun to be reported [46, 47] and used more widely outside specialist groups [48].

These estimates should be shared with patients who want to understand their prognosis and wish to know their likelihood of remaining recurrence‐free in the future [49]. Our results, revealing clear differences in MR risk between stages and BC surrogate subtypes, may provide important information on crucial points in the follow‐up [50]. For HER2‐positive and TN disease, recurrences most often occur in the early years after diagnosis, suggesting that follow‐up for these patients should primarily focus on the first few years [51]. For HR+/HER2‐ disease, MR is also common many years after diagnosis [52, 53], an observation that deserves consideration in the follow‐up plan. This information may also have implications for patient‐tailored treatment.

Our findings on the conditional risk of MR are particularly useful to understand breast cancer complexity [54] and for patient risk stratification. These conditional estimates have not only an impact on clinical practice but also on the social and psychological aspects that patients have to deal with, and are also essential to reduce their discrimination [55, 56]. Finally, the presented methodology offers the opportunity to support appropriate healthcare policies, allowing estimation of MR risk from population‐based cancer registry data at wider (e.g., European) levels, as well as for other cancer types.

## 5 Conclusion

This study provides the first population‐based estimates of MR up to 15 years since BC diagnosis by stage and molecular profile for Italian women. These results, shown in terms of cumulative and conditional risks for women alive and recurrence‐free after 5 and 10 years, may help in tailoring follow‐up through informative risk stratification.

## Author Contributions

Luigino Dal Maso and Stefano Guzzinati drafted the study protocol. The other authors revised the study protocol, collected the data, and prepared the cleaned data for the study database (Fabiola Giudici, Sara De Vidi, Federica Toffolutti, Silvia Francisci, Angela B Mariotto, Alessandra Ravaioli, Ettore Bidoli, Fabio Falcini, Antonella Puppo, Lucia Mangone, Maria Michiara, Mario Fusco, Anna Clara Fanetti).

Fabiola Giudici, Sara De Vidi, Stefano Guzzinati, Federica Toffolutti, and Luigino Dal Maso designed the study and did the statistical analyses. Silvia Francisci, Angela B Mariotto, Alessandra Ravaioli, Laura Botta, Roberta De Angelis, and Riccardo Capocaccia contributed to the validation of statistical models and revised the statistical analyses. Diego Serraino specifically discussed the assumptions and clinical implications of the estimates of metastatic recurrence.

The first draft of the manuscript was written by Fabiola Giudici and Luigino Dal Maso.

All authors contributed to the interpretation of the study results, critically commented on the contents of preliminary versions of the manuscript, and approved the submitted version.

## Funding

The work leading to this manuscript was supported by the AIRC IG [grant number 28893] and by the Italian Ministry of Health (Ricerca Corrente). The funding sources had no involvement in the study design; in the collection, analysis, and interpretation of data; in the writing of the report; and in the decision to submit the article for publication.

## Ethics Statement

According to the Italian legislation [57] population‐based cancer registries collect pseudonymised personal data for surveillance purposes that do not need the collection of explicit individual consent, without any direct or indirect intervention on patients; therefore, the approval of a research ethics committee was not required.

## Conflicts of Interest

The authors declare no conflicts of interest.

## Supporting information


**Data S1:** cam471492‐sup‐0001‐supinfo.docx.

## Data Availability

The data that supports the findings of this study are described in the Section 2 of this article. Further information is available from the corresponding author upon request.
