# Supplementary material for: Metastatic Recurrence of Breast Cancer by Stage and Molecular Profile: A Population‐Based Study Among Italian Women
Source: Cancer Med. 2026 Jan 4;15(1):e71492. doi: 10.1002/cam4.71492 (PMC12766157; doi:10.1002/cam4.71492)
Supplement: Supplementary file 1 — Data S1: cam471492‐sup‐0001‐supinfo.docx. [file CAM4-15-e71492-s001.docx]

**Figure S1: Model of the pathway between diagnosis, metastatic recurrence, and death**


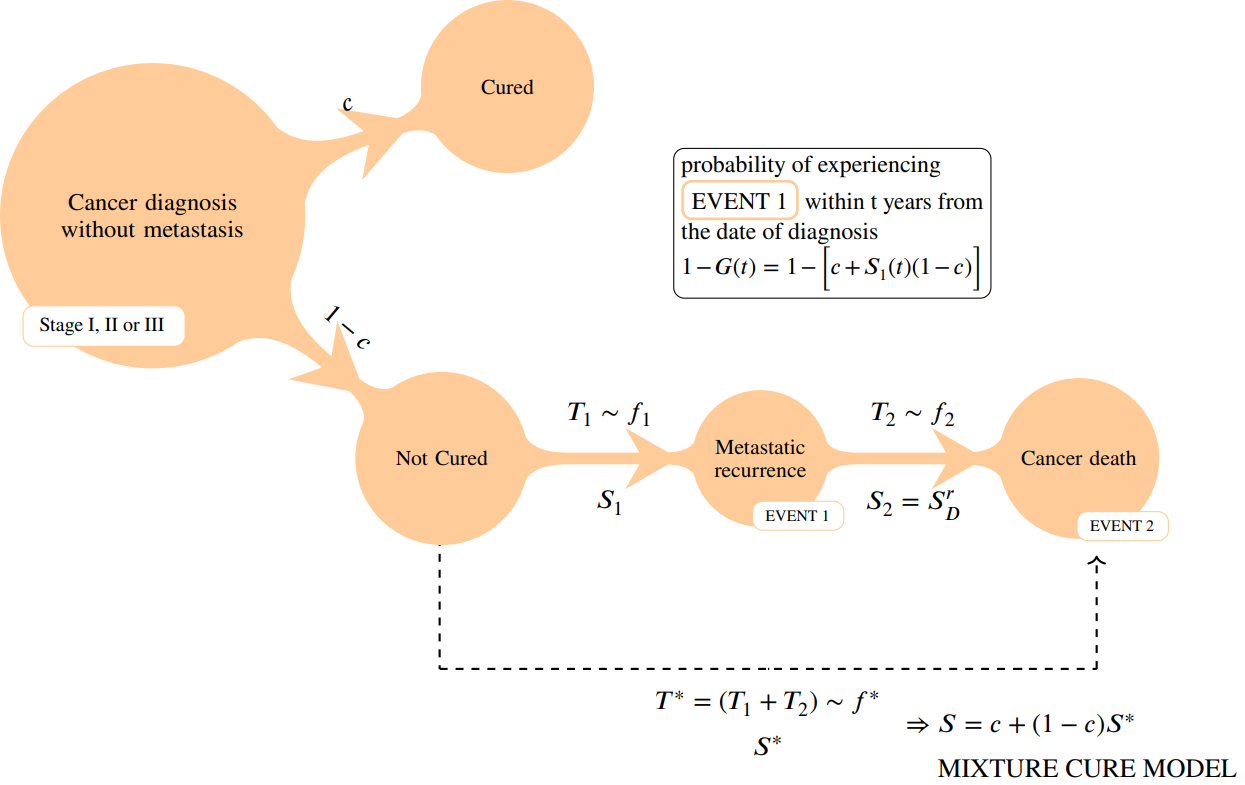


After cancer diagnosis, a proportion c of patients is not at risk of dying of their cancer (cured) and a proportion (1-c) is at risk of dying of their cancer (not cured). Adapted from Mariotto et al., 2018 (10).

*c* = cured patients; 1-*c* = not cured patients; $f_{i}$=probability density function for $T_{i}$ times, for events (*i*) 1 and 2; $f^{*}$=probability density function for $T^{*}$ times; $S_{1}$=survival function to metastasis for uncured patients; $S_{2}$=survival function from metastasis to cancer death for uncured patients, estimated from de novo metastatic breast cancer and adjusted for a rate ratio factor comparing de novo versus metachronous metastatic breast cancer (22); $S^{*}$=survival function for uncured patients, estimated from a mixture cure model by“subtraction” methods using S_2_ and S*.

**Figure S2:** **Flowchart showing the models and software used to calculate the risk of metastatic recurrence for breast cancer patients. Italian Cancer Registries, 1997-2017, follow-up 2018**

**
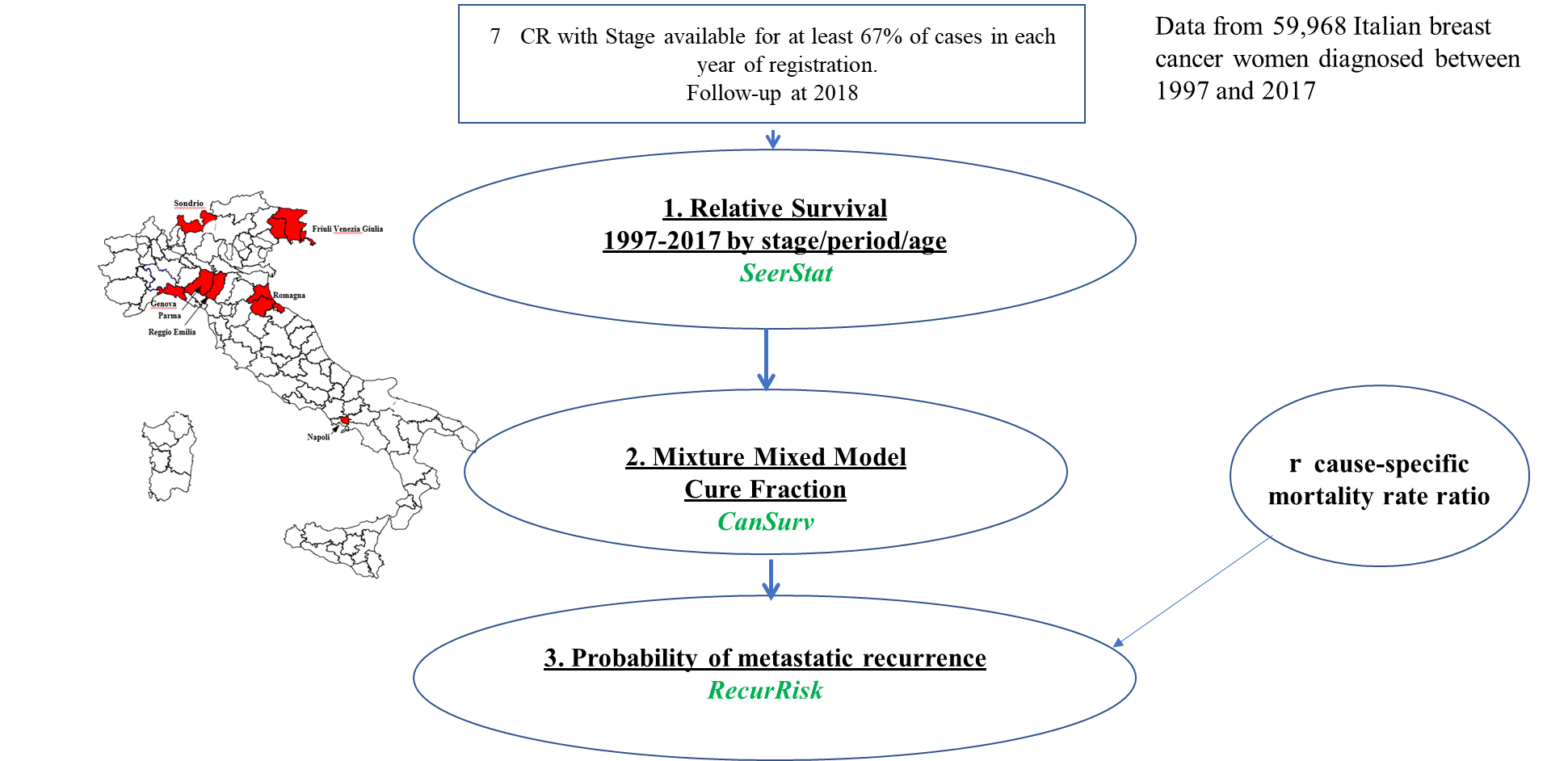
**

**Table S1: Breast cancer cases in Italian cancer registries contributing to the present study by stage^1^, 1997-2017. Ages 15-74.**

|  |  | | **Stages** | | | | | | | | | | | |
| --- | --- | --- | --- | --- | --- | --- | --- | --- | --- | --- | --- | --- | --- | --- |
| **Registry** | **Period** | | **All but missing** | **I** | | **II** | | **III** | | **IV** | | **Missing** | |  |
|  |  | | **N** | **N** | **%** | **N** | **%** | **N** | **%** | **N** | **%** | **N** | **%** |  |
| **Friuli VG^2^** | | 1997-2017 | 17,001 | 8,626 | 51% | 5,603 | 33% | 1,968 | 12% | 804 | 5% | 2,080 | 11% |  |
| **Romagna^2^** | | 1997-2017 | 14,047 | 7,766 | 55% | 4,476 | 32% | 1,356 | 10% | 449 | 3% | 1,211 | 8% |  |
| **Genova** | | 1997-2016 | 10,997 | 5,435 | 49% | 4,025 | 37% | 1,391 | 13% | 146 | 1% | 1,793 | 14% |  |
| **Reggio Emilia^2^** | | 1997-2017 | 6,455 | 3,433 | 53% | 2,115 | 33% | 726 | 11% | 181 | 3% | 583 | 8% |  |
| **Parma^2^** | | 1997-2017 | 5,651 | 2,983 | 53% | 1,805 | 32% | 696 | 12% | 167 | 3% | 521 | 8% |  |
| **Napoli** | | 1997-2017 | 3,952 | 1,504 | 38% | 1,691 | 43% | 586 | 15% | 171 | 4% | 1,141 | 22% |  |
| **Sondrio** | | 1998-2016 | 1,865 | 871 | 47% | 700 | 38% | 184 | 10% | 110 | 6% | 226 | 11% |  |
|  |  | |  |  |  |  |  |  |  |  |  |  |  |  |
| **Total^3^** |  | | **59,968** | **30,618** | **51%** | **20,415** | **34%** | **6,907** | **12%** | **2,028** | **3%** | **7,555** | **11%** |  |

^1^ Registries were included when stage information was available for at least 67% of the patients in each calendar year of registration.

**^2^** Registries contributing also to analyses by surrogate molecular subtype. Cases included are reported in Supplementary Table 2.

**^3^** Among all incident cases of BC (90,280), women older than 75 years (n=22,757, 25.2%) and with missing stages (n=7,555, 11.2%) were excluded.

**Table S2: Breast cancer cases in Italian cancer registries contributing to the present study by surrogate molecular profile^1^, 2003-2012. Ages 15-74.**

|  | |  | |  | **Surrogate Molecular Profile** | | | | | |  |  |
| --- | --- | --- | --- | --- | --- | --- | --- | --- | --- | --- | --- | --- |
| **Registry** | **Period** | | **All but missing** | | **HR+/HER2-** | | **HER2+** | | **Triple Negative** | | **Missing** | |
|  |  | | **N** | | **N** | **%** | **N** | **%** | **N** | **%** | **N** | **%** |
| **Friuli Venezia Giulia** | 2003-2012 | | 7,194 | | 5,322 | 74.0% | 1,209 | 16.8% | 663 | 9.2% | 1,436 | 16.6% |
| **Parma** | 2003-2012 | | 2,278 | | 1,776 | 78.0% | 287 | 12.6% | 215 | 9.4% | 449 | 16.5% |
| **Romagna** | 2003-2012 | | 6,403 | | 4,938 | 77.1% | 993 | 15.5% | 472 | 7.4% | 294 | 4.4% |
| **Reggio Emilia** | 2003-2012 | | 2,698 | | 2,102 | 77.9% | 428 | 15.9% | 168 | 6.2% | 338 | 11.1% |
|  |  | |  | |  |  |  |  |  |  |  |  |
| **Total** |  | | **18,573** | | **14,138** | **76.1%** | **2,917** | **15.7%** | **1518** | **8.2%** | **2,517** | **11.9%** |

^1^ Registries were included when surrogate molecular profile information was available for at least 67% of the patients after 2003.

**Table S3: Breast cancer cases in Italian cancer registries contributing to the present study^1^ by stage, age at diagnosis, and surrogate molecular profile.**

|  | **Age Groups (years)** | | | | | | | | | | | | | | | | | | | | |
| --- | --- | --- | --- | --- | --- | --- | --- | --- | --- | --- | --- | --- | --- | --- | --- | --- | --- | --- | --- | --- | --- |
| **Stage** | **15-74** | | | | | | | **15-54** | | | | | | | **55-74** | | | | | | |
|  | **HR+/HER2-** | | | **HER2+** | | **TN** | | **HR+/HER2-** | | **HER2+** | | **TN** | | **HR+/HER2-** | | | **HER2+** | | **TN** | | |
|  | **N** | | **%** | **N** | **%** | **N** | **%** | **N** | **%** | **N** | **%** | **N** | **%** | **N** | | **%** | **N** | **%** | **N** | **%** |  |
| **I** | | **7,804** | **55.2%** | **1,179** | **40.4%** | **620** | **40.8%** | 2,738 | 51.9% | 520 | 38.8% | 244 | 37.3% | 5,066 | | 57.1% | 659 | 41.8% | 376 | 43.6% |  |
| **II** | | **4,390** | **31.1%** | **1,050** | **36.0%** | **601** | **39.6%** | 1,771 | 33.6% | 509 | 38.0% | 288 | 44.0% | 2,619 | | 29.5% | 541 | 34.3% | 313 | 36.3% |  |
| **III** | | **1,570** | **11.1%** | **521** | **17.9%** | **211** | **13.9%** | 643 | 12.2% | 244 | 18.2% | 89 | 13.6% | 927 | | 10.5% | 277 | 17.6% | 122 | 14.1% |  |
| **IV** | | **374** | **2.6%** | **167** | **5.7%** | **86** | **5.7%** | 121 | 2.3% | 66 | 4.9% | 34 | 5.2% | 253 | | 2.9% | 101 | 6.4% | 52 | 6.0% |  |
| **All Stages** | | **14,138** | **100.0%** | **2,917** | **100.0%** | **1,518** | **100.0%** | 5,273 | 100.0% | 1,339 | 100.0% | 655 | 100.0% | 8,865 | | 100.0% | 1,578 | 100.0% | 863 | 100.0% |  |

^1^ Breast cancer cases aged 15-74, diagnosed in 2003-2012 and followed up until 2018.

**Table S4: Number of patients diagnosed in 1997–2017 and followed up until 2018 and breast cancer relative survival (RS) in percentage by stage, age, and period of diagnosis^1^.**

|  |  |  | **Relative Survival (RS)** | | | | |
| --- | --- | --- | --- | --- | --- | --- | --- |
| **Stage** | **Age** | **N at t_0_** | **5-yr RS** | **N at t_9_** | **10-yr RS** | **N at t _14_** | **15-yr RS** |
|  |  |  |  |  |  |  |  |
| **All** | **15-44** | 8,369 | 91.2% | 3926 | 84.2% | 1893 | 78.2% |
|  | **45-54** | 15,735 | 93.7% | 6985 | 88.8% | 3596 | 85.0% |
|  | **55-64** | 17,247 | 93.0% | 8124 | 86.1% | 3993 | 81.2% |
|  | **65-74** | 18,541 | 91.4% | 7159 | 84.5% | 2958 | 78.7% |
|  |  |  |  |  |  |  |  |
| **I** | **15-44** | 3,525 | 98.4% | 1797 | 95.6% | 929 | 92.4% |
|  | **45-54** | 8,077 | 99.2% | 3692 | 97.7% | 1958 | 96.1% |
|  | **55-64** | 9,292 | 99.4% | 4625 | 97.1% | 2293 | 94.4% |
|  | **65-74** | 9,698 | 100.% | 2988 | 99.0% | 1671 | 96.0% |
|  |  |  |  |  |  |  |  |
| **II** | **15-44** | 3,393 | 92.5% | 1687 | 84.1% | 805 | 75.8% |
|  | **45-54** | 5,522 | 94.9% | 2655 | 88.6% | 1407 | 83.6% |
|  | **55-64** | 5,512 | 93.8% | 2791 | 85.1% | 1432 | 78.3% |
|  | **65-74** | 5,979 | 92.8% | 2549 | 82.7% | 1073 | 75.1% |
|  |  |  |  |  |  |  |  |
| **III** | **15-44** | 1,186 | 79.6% | 417 | 65.8% | 154 | 58.1% |
|  | **45-54** | 1,725 | 81.3% | 613 | 68.1% | 226 | 58.5% |
|  | **55-64** | 1,863 | 80.7% | 671 | 60.2% | 256 | 50.3% |
|  | **65-74** | 2,130 | 72.4% | 579 | 54.0% | 207 | 42.7% |
|  |  |  |  |  |  |  |  |
| **IV** | **15-44** | 270 | 33.5% | 25 | 20.4% | 5 | 13.3% |
|  | **45-54** | 419 | 27.5% | 25 | 13.4% | 5 | 7.7% |
|  | **55-64** | 589 | 26.7% | 38 | 12.8% | 12 | 9.4% |
|  | **65-74** | 747 | 23.8% | 43 | 11.4% | 7 | 5.7% |
|  |  |  |  |  |  |  |  |

^1^The number of patients N for a time from diagnosis equal 5, 10, and 15 years represents respectively the number of cases at diagnosis and the number of cases alive after 9 years and 14 years since diagnosis.

**Table S5. Risk of metastatic recurrence for Italian breast cancer patients diagnosed in 1997–2017 by stage, period, and age group.**

|  |  |  | **Age groups (years)** | | | | |
| --- | --- | --- | --- | --- | --- | --- | --- |
| **Stage** | **Period of Diagnosis** | **Years since diagnosis** | **15-44** | **45-54** | **55-64** | **65-74** | **15-74** |
|  |  |  |  |  |  |  |  |
| **I** | **1997-2006** | **≤ 5** | 3.8% | 1.9% | 2.5% | 1.6% | **2.1%** |
|  |  | **≤ 10** | 7.7% | 4.0% | 5.4% | 3.5% | **4.9%** |
|  |  | **≤ 15** | 10.7% | 5.7% | 7.7% | 5.0% | **7.2%** |
|  |  |  |  |  |  |  |  |
|  | **2007-2017** | **≤ 5** | 2.7% | 1.4% | 1.8% | 1.1% | **1.0%** |
|  |  | **≤ 10** | 5.4% | 2.8% | 3.7% | 2.3% | **2.1%** |
|  |  | **≤ 15** | 7.3% | 3.9% | 5.3% | 3.3% | **3.0%** |
|  |  |  |  |  |  |  |  |
|  |  |  |  |  |  |  |  |
| **II** | **1997-2006** | **≤ 5** | 16.6% | 10.5% | 13.4% | 14.7% | **13.5%** |
|  |  | **≤ 10** | 26.1% | 17.6% | 22.3% | 25.1% | **22.4%** |
|  |  | **≤ 15** | 30.8% | 21.4% | 27.2% | 30.9% | **27.1%** |
|  |  |  |  |  |  |  |  |
|  | **2007-2017** | **≤ 5** | 10.5% | 6.8% | 8.5% | 9.2% | **8.4%** |
|  |  | **≤ 10** | 16.0% | 10.6% | 13.6% | 15.3% | **13.5%** |
|  |  | **≤ 15** | 18.6% | 12.4% | 16.2% | 18.7% | **16.0%** |
|  |  |  |  |  |  |  |  |
|  |  |  |  |  |  |  |  |
| **III** | **1997-2006** | **≤ 5** | 37.8% | 32.8% | 37.5% | 42.5% | **38.2%** |
|  |  | **≤ 10** | 48.1% | 44.4% | 50.8% | 59.0% | **51.4%** |
|  |  | **≤ 15** | 50.6% | 48.2% | 55.1% | 64.6% | **55.6%** |
|  |  |  |  |  |  |  |  |
|  | **2007-2017** | **≤ 5** | 30.2% | 27.7% | 31.4% | 36.1% | **31.0%** |
|  |  | **≤ 10** | 37.2% | 35.1% | 40.7% | 49.0% | **40.1%** |
|  |  | **≤ 15** | 38.5% | 36.8% | 43.3% | 53.2% | **42.7%** |
|  |  |  |  |  |  |  |  |
|  |  |  |  |  |  |  |  |
| **I-III** | **1997-2006** | **≤ 5** | 14.6% | 8.8% | 10.5% | 11.4% | **11.0%** |
|  |  | **≤ 10** | 22.1% | 14.2% | 16.8% | 18.7% | **17.5%** |
|  |  | **≤ 15** | 25.3% | 16.8% | 19.9% | 22.4% | **20.6%** |
|  |  |  |  |  |  |  |  |
|  | **2007-2017** | **≤ 5** | **9.9%** | **6.1%** | **7.1%** | **7.4%** | **7.0%** |
|  |  | **≤ 10** | **14.5%** | **9.2%** | **10.8%** | **11.9%** | **10.7%** |
|  |  | **≤ 15** | **16.3%** | **10.5%** | **12.6%** | **14.1%** | **12.3%** |
|  |  |  |  |  |  |  |  |

**Table S6: Risk of metastatic recurrence for Italian breast cancer patients diagnosed in 2003–2012 by stage, age group, and surrogate molecular profile.**

|  | | | | | | | | | | | | |
| --- | --- | --- | --- | --- | --- | --- | --- | --- | --- | --- | --- | --- |
|  |  | **Age Groups (years)** | | | | | | | | | |  |
| **Stage** | **Years since diagnosis** | **15-54** | | | |  | | **55-74** | | | |  |
|  |  | **HR+/HER2-** | **HER2+** | **TN** |  | | **HR+/HER2-** | | **HER2+** | **TN** |  |  |
| **I** | **≤ 5** | 0.9% | 2.6% | 6.3% |  | | 0.7% | | 2.4% | 6.5% |  |  |
|  | **≤ 10** | 2.8% | 5.7% | 7.4% |  | | 2.4% | | 5.5% | 7.7% |  |  |
|  | **≤ 15** | 4.6% | 6.8% | 7.7% |  | | 4.3% | | 6.8% | 8.0% |  |  |
|  |  |  |  |  |  | |  | |  |  |  |  |
| **II** | **≤ 5** | 8.3% | 11.7% | 14.9% |  | | 8.4% | | 13.0% | 18.5% |  |  |
|  | **≤ 10** | 13.7% | 15.1% | 16.3% |  | | 15.3% | | 18.1% | 20.4% |  |  |
|  | **≤ 15** | 15.1% | 15.9% | 16.7% |  | | 17.9% | | 19.6% | 20.9% |  |  |
|  |  |  |  |  |  | |  | |  |  |  |  |
| **III** | **≤ 5** | 30.1% | 32.1% | 45.1% |  | | 32.6% | | 37.8% | 58.5% |  |  |
|  | **≤ 10** | 40.1% | 41.1% | 48.5% |  | | 47.6% | | 51.4% | 63.2% |  |  |
|  | **≤ 15** | 43.5% | 44.5% | 49.6% |  | | 54.1% | | 57.1% | 64.8% |  |  |
|  |  |  |  |  |  | |  | |  |  |  |  |
| **I-III** | **≤ 5** | 6.5% | 12.2% | 16.0% |  | | 6.1% | | 12.7% | 18.3% |  |  |
|  | **≤ 10** | 11.6% | 15.7% | 17.6% |  | | 11.8% | | 17.4% | 20.3% |  |  |
|  | **≤ 15** | 14.1% | 16.8% | 18.1% |  | | 15.2% | | 19.1% | 20.9% |  |  |

**Table S7. Conditional probabilities of metastatic recurrence for Italian breast cancer patients diagnosed in 2003–2012 by stage, age group, and surrogate molecular profile.**

|  |  | Age Groups (years) | | | | | | |
| --- | --- | --- | --- | --- | --- | --- | --- | --- |
| Stage | **Years since diagnosis** | **15-54** | | |  | **55-74** | | |
|  |  | **HR+/HER2-** | **HER2+** | **TN** |  | **HR+/HER2-** | **HER2+** | **TN** |
| I | **0 to 5** | 0.9% | 2.6% | 6.3% |  | 0.7% | 2.4% | 6.5% |
|  | **5 to 10** | 1.9% | 3.1% | 1.2% |  | 1.8% | 3.2% | 1.3% |
|  | **10 to 15** | 1.9% | 1.2% | 0.3% |  | 1.9% | 1.4% | 0.3% |
|  |  |  |  |  |  |  |  |  |
| II | **0 to 5** | 8.3% | 11.7% | 14.9% |  | 8.4% | 13.0% | 18.5% |
|  | **5 to 10** | 5.9% | 3.9% | 1.7% |  | 7.5% | 5.9% | 2.4% |
|  | **10 to 15** | 1.6% | 0.9% | 0.5% |  | 3.1% | 1.8% | 0.7% |
|  |  |  |  |  |  |  |  |  |
| III | **0 to 5** | 30.1% | 32.1% | 45.1% |  | 32.6% | 37.8% | 58.5% |
|  | **5 to 10** | 14.3% | 13.2% | 6.0% |  | 22.2% | 21.9% | 11.2% |
|  | **10 to 15** | 5.6% | 5.8% | 2.3% |  | 12.5% | 11.6% | 4.3% |
|  |  |  |  |  |  |  |  |  |
| I-III | **0 to 5** | **6.5%** | **12.2%** | **16.0%** |  | **6.1%** | **12.7%** | **18.3%** |
|  | **5 to 10** | **5.5%** | **4.0%** | **1.9%** |  | **6.1%** | **5.4%** | **2.5%** |
|  | **10 to 15** | **2.8%** | **1.3%** | **0.6%** |  | **3.8%** | **2.1%** | **0.8%** |

**Table S8. Sensitivity analysis: risk of metastatic recurrence for Italian breast cancer patients diagnosed in 2007-2017 by stage and selected age groups (15-44 years and 65-74 years) according to different r values.**

|  |  | Age 15-44 | | | |  | Age 65-74 | | | |
| --- | --- | --- | --- | --- | --- | --- | --- | --- | --- | --- |
|  | **Years since diagnosis** | **r=1.00** | **r=1.33*** | **r=1.50** | **r=1.70** |  | **r=1.00** | **r=1.33*** | **r=1.50** | **r=1.70** |
|  |  |  |  |  |  |  |  |  |  |  |
| Stage I | **≤ 5** | 3.3% | 2.7% | 2.6% | 2.4% |  | 1.3% | 1.1% | 1.0% | 1.0% |
|  | **≤ 10** | 6.0% | 5.4% | 5.1% | 4.9% |  | 2.5% | 2.3% | 2.2% | 2.2% |
|  | **≤ 15** | 7.9% | 7.3% | 7.1% | 7.0% |  | 3.5% | 3.3% | 3.2% | 3.2% |
|  |  |  |  |  |  |  |  |  |  |  |
|  |  |  |  |  |  |  |  |  |  |  |
| Stage II | **≤ 5** | 12.3% | 10.5% | 9.8% | 9.3% |  | 10.5% | 9.2% | 8.7% | 8.3% |
|  | **≤ 10** | 17.6% | 16.0% | 15.5% | 15.0% |  | 16.4% | 15.3% | 14.9% | 14.6% |
|  | **≤ 15** | 19.5% | 18.6% | 18.2% | 17.9% |  | 19.3% | 18.7% | 18.4% | 18.2% |
|  |  |  |  |  |  |  |  |  |  |  |
|  |  |  |  |  |  |  |  |  |  |  |
| Stage III | **≤ 5** | 35.1% | 30.2% | 28.6% | 27.1% |  | 40.7% | 36.1% | 34.5% | 33.0% |
|  | **≤ 10** | 38.5% | 37.2% | 36.3% | 35.5% |  | 51.6% | 49.0% | 48.1% | 47.3% |
|  | **≤ 15** | 38.5% | 38.5% | 38.2% | 37.9% |  | 54.2% | 53.2% | 52.9% | 52.6% |
|  |  |  |  |  |  |  |  |  |  |  |
|  |  |  |  |  |  |  |  |  |  |  |
| Stage I-III | **≤ 5** | 11.6% | 9.9% | 9.3% | 8.7% |  | 8.5% | 7.4% | 7.1% | 6.7% |
|  | **≤ 10** | 15.8% | 14.5% | 14.0% | 13.6% |  | 12.7% | 11.9% | 11.6% | 11.4% |
|  | **≤ 15** | 16.9% | 16.3% | 16.0% | 15.8% |  | 14.6% | 14.1% | 14.0% | 13.8% |

*****We use a mortality hazard ratio r= 1.33 (De Maar et al, 2023) to relate the survival after metastatic recurrence to the survival from de novo metastatic diagnosis. A factor higher than 1 represents higher probability of death from breast cancer after metastatic recurrence than after a de novo metastatic breast cancer. (r=1.00 same probability of death, that is not supported by real word data)

**Table S9. Sensitivity analysis: risk of metastatic recurrence for Italian breast cancer patients aged 15-54 years and diagnosed in 2003–2012 by stage and surrogate molecular profile according to different r values**

|  |  | HR+/HER2- | | | |  | HER2+ | | | |  | TN | | | |
| --- | --- | --- | --- | --- | --- | --- | --- | --- | --- | --- | --- | --- | --- | --- | --- |
| Stage | **Years since diagnosis** | **r=1.00** | **r=1.33*** | **r =1.50** | **r=1.70** |  | **r=1.00** | **r=1.33*** | **r =1.50** | **r=1.70** |  | **r=1.00** | **r=1.33*** | **r =1.50** | **r=1.70** |
| I | **≤ 5** | 1.1% | 0.9% | 0.8% | 0.7% |  | 3.1% | 2.6% | 2.4% | 2.2% |  | 6.9% | 6.3% | 6.1% | 6.0% |
|  | **≤ 10** | 3.3% | 2.8% | 2.6% | 2.4% |  | 6.3% | 5.7% | 5.4% | 5.2% |  | 7.5% | 7.4% | 7.4% | 7.4% |
|  | **≤ 15** | 5.2% | 4.6% | 4.4% | 4.2% |  | 7.1% | 6.8% | 6.6% | 6.5% |  | 7.7% | 7.7% | 7.7% | 7.7% |
|  |  |  |  |  |  |  |  |  |  |  |  |  |  |  |  |
| II | **≤ 5** | 10.2% | 8.3% | 7.6% | 7.1% |  | 13.8% | 11.7% | 10.9% | 10.2% |  | 15.8% | 14.9% | 14.6% | 14.3% |
|  | **≤ 10** | 15.5% | 13.7% | 13.1% | 12.5% |  | 16.2% | 15.1% | 14.7% | 14.4% |  | 16.5% | 16.3% | 16.3% | 16.2% |
|  | **≤ 15** | 16.1% | 15.1% | 14.8% | 14.5% |  | 16.3% | 15.9% | 15.7% | 15.6% |  | 16.8% | 16.7% | 16.7% | 16.7% |
|  |  |  |  |  |  |  |  |  |  |  |  |  |  |  |  |
| III | **≤ 5** | 36.3% | 30.1% | 28.0% | 26.1% |  | 37.4% | 32.1% | 30.3% | 28.6% |  | 47.0% | 45.1% | 44.5% | 43.9% |
|  | **≤ 10** | 44.5% | 40.1% | 38.6% | 37.2% |  | 44.0% | 41.1% | 40.1% | 39.2% |  | 48.8% | 48.5% | 48.3% | 48.2% |
|  | **≤ 15** | 45.9% | 43.5% | 42.6% | 41.9% |  | 45.9% | 44.5% | 44.1% | 43.6% |  | 49.8% | 49.6% | 49.6% | 49.6% |
|  |  |  |  |  |  |  |  |  |  |  |  |  |  |  |  |
| I-III | **≤ 5** | 7.9% | 6.5% | 6.0% | 5.5% |  | 14.3% | 12.2% | 11.5% | 10.8% |  | 16.8% | 16.0% | 15.7% | 15.4% |
|  | **≤ 10** | 13.2% | 11.6% | 11.0% | 10.5% |  | 16.8% | 15.7% | 15.3% | 14.9% |  | 17.7% | 17.6% | 17.5% | 17.5% |
|  | **≤ 15** | 15.2% | 14.1% | 13.7% | 13.3% |  | 17.3% | 16.8% | 16.6% | 16.5% |  | 18.2% | 18.1% | 18.1% | 18.1% |

*****We use a mortality hazard ratio r= 1.33 (De Maar et al, 2023) to relate the survival after metastatic recurrence to the survival from de novo metastatic diagnosis. A factor higher than 1 represents higher probability of death from breast cancer after metastatic recurrence than after a de novo metastatic breast cancer. (r=1.00 same probability of death, that is not supported by real word data)

**Table S10. Sensitivity analysis: risk of metastatic recurrence for Italian breast cancer patients aged 55-74 years and diagnosed in 2003–2012 by stage and surrogate molecular profile according to different r values**

|  | |  | HR+/HER2- | | | |  | HER2+ | | | |  | TN | | | |
| --- | --- | --- | --- | --- | --- | --- | --- | --- | --- | --- | --- | --- | --- | --- | --- | --- |
| Stage | **Years since diagnosis** | | **r=1.00** | **r=1.33** | **r =1.50** | **r=1.70** |  | **r=1.00** | **r=1.33** | **r =1.50** | **r=1.70** |  | **r=1.00** | **r=1.33** | **r =1.50** | **r=1.70** |
| I | | **≤ 5** | 0.9% | 0.7% | 0.7% | 0.6% |  | 2.8% | 2.4% | 2.8% | 2.1% |  | 7.0% | 6.5% | 7.0% | 6.1% |
|  |  | **≤ 10** | 2.8% | 2.4% | 2.3% | 2.2% |  | 6.0% | 5.5% | 6.0% | 5.1% |  | 7.9% | 7.7% | 7.9% | 7.7% |
|  |  | **≤ 15** | 4.7% | 4.3% | 4.1% | 4.0% |  | 7.1% | 6.8% | 7.1% | 6.6% |  | 8.1% | 8.0% | 8.1% | 8.0% |
|  | |  |  |  |  |  |  |  |  |  |  |  |  |  |  |  |
| II | | **≤ 5** | 10.1% | 8.4% | 7.8% | 7.3% |  | 15.2% | 13.0% | 15.2% | 11.6% |  | 19.5% | 18.5% | 19.5% | 17.8% |
|  |  | **≤ 10** | 16.9% | 15.3% | 14.7% | 14.2% |  | 19.2% | 18.1% | 19.2% | 17.4% |  | 20.6% | 20.4% | 20.6% | 20.3% |
|  |  | **≤ 15** | 18.8% | 17.9% | 17.6% | 17.3% |  | 20.0% | 19.6% | 20.0% | 19.3% |  | 21.0% | 20.9% | 21.0% | 20.9% |
|  | |  |  |  |  |  |  |  |  |  |  |  |  |  |  |  |
| III | | **≤ 5** | 38.4% | 32.6% | 30.6% | 28.7% |  | 43.4% | 37.8% | 43.4% | 34.1% |  | 60.8% | 58.5% | 60.8% | 57.0% |
|  |  | **≤ 10** | 51.7% | 47.6% | 46.1% | 44.8% |  | 54.4% | 51.4% | 54.4% | 49.4% |  | 63.6% | 63.2% | 63.6% | 62.9% |
|  |  | **≤ 15** | 56.4% | 54.1% | 53.3% | 52.6% |  | 58.5% | 57.1% | 58.5% | 56.1% |  | 64.9% | 64.8% | 64.9% | 64.7% |
|  | |  |  |  |  |  |  |  |  |  |  |  |  |  |  |  |
| I-III | | **≤ 5** | 7.3% | 6.1% | 5.7% | 5.3% |  | 14.6% | 12.7% | 14.6% | 11.4% |  | 19.2% | 18.3% | 19.2% | 17.6% |
|  |  | **≤ 10** | 13.2% | 11.8% | 11.4% | 10.9% |  | 18.4% | 17.4% | 18.4% | 16.7% |  | 20.5% | 20.3% | 20.5% | 20.2% |
|  |  | **≤ 15** | 16.1% | 15.2% | 14.8% | 14.5% |  | 19.5% | 19.1% | 19.5% | 18.8% |  | 21.0% | 20.9% | 21.0% | 20.9% |

*****We use a mortality hazard ratio r= 1.33 (De Maar et al, 2023) to relate the survival after metastatic recurrence to the survival from de novo metastatic diagnosis. A factor higher than 1 represents higher probability of death from breast cancer after metastatic recurrence than after a de novo metastatic breast cancer. (r=1.00 same probability of death, that is not supported by real word data)

**Figure S3: Five-year conditional relative survival (5-yr CRS) and model-based estimates for Italian breast cancer patients diagnosed in 2003–2012 by molecular profile, stage and age.**

**HR+/HER2−**

**
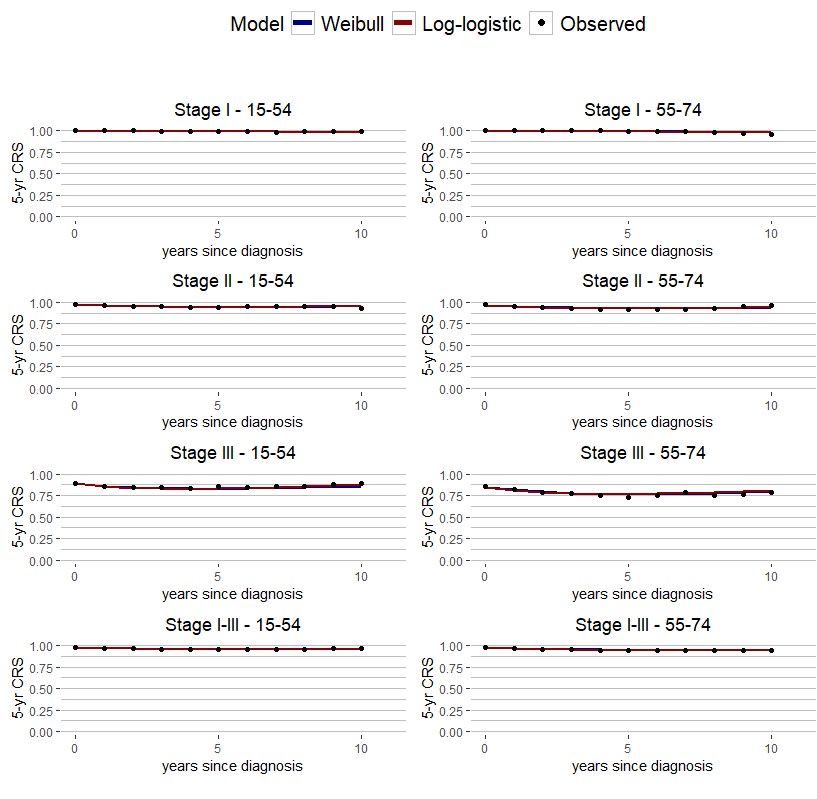
**

**Continued**

**continues**

**HER2+**

**
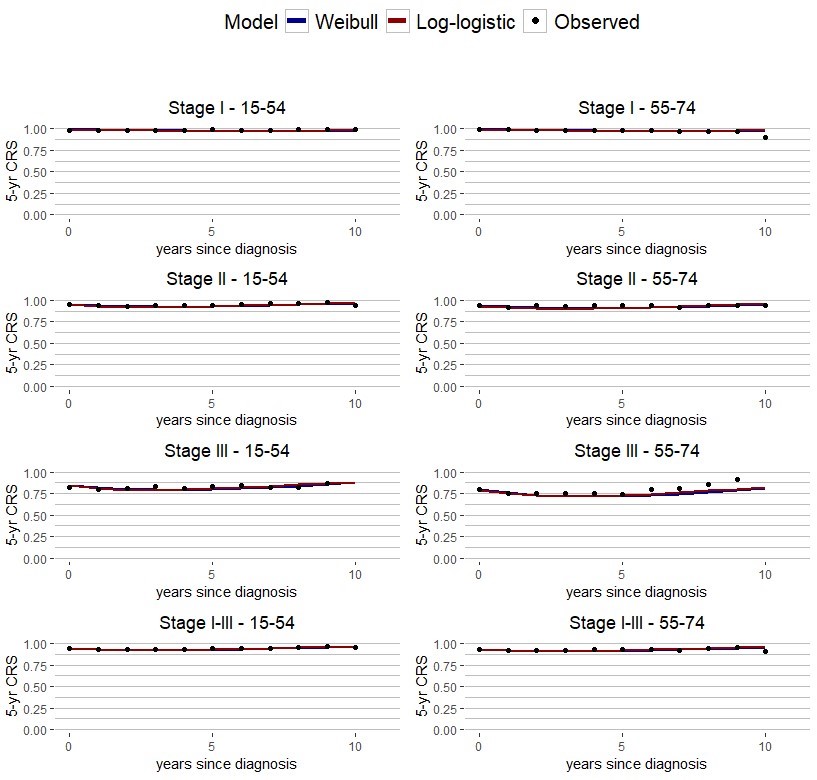
**

**Continued**

**continues**

**Triple Negative**

**
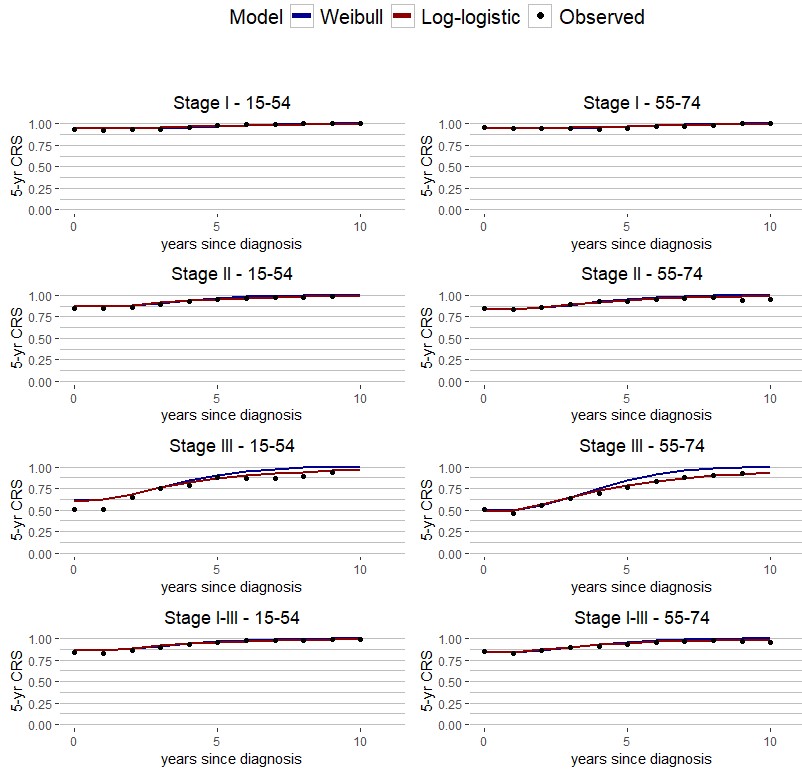
**

**Supporting Information Methodological Material**

## **Model to estimate the risk of developing distant metastatic breast cancer**

The model allows the estimation of the probability of progression or recurrence to distant metastatic cancer within *t* years from diagnosis, after a diagnosis at an earlier stage of the disease. Therefore, this probability includes both distant metastatic recurrences, i.e., the detection of distant metastasis after being disease-free following treatment, and progression to distant metastasis without ever being disease-free.

To obtain this probability, the method assumes that recurrence or progression to distant metastatic disease must happen before a cancer death. Also, it describes the survival time for those not cured in the mixture cure model, $T^{*}$, as the sum of the time from diagnosis to the development of distant metastatic cancer (T₁) and the time from the event of distant metastasis to cancer death (T₂) (**Figure S1**). Because T₁ and T₂ are assumed as independent random variables, $T^{*}$ is a convolution of T₁ and T₂.

It has been shown (10) that assuming T₂ exponential, there is an analytical derivation of the distribution of T₁ , $f_{1}$. This leads to a survival function of T₁ that depends only on the distribution of $T^{*}$, and the parameter of the exponential distribution that was assumed for T₂. This means that if $T^{*}$ satisfies the initial hypothesis, i.e. is a convolution of T₁ and T₂, we can use only the distribution of $T^{*}$ and the parameter of the exponential to obtain the unknown distribution of T₁. The distribution of $T^{*}$ is the distribution for those not cured in the mixture cure models. It is common to consider $T^{*}$ following a Weibull and Log-Logistic distribution, but we underline that not all distributions for $T^{*}$ allow this to be the result of the convolution of an exponential distribution and a hypothetical random variable for T₁, so we need to pay attention to the analytical result of the distribution of T₁. For example, if we want to use Weibull or Log-Logistic distribution for $T^{*}$, we have to verify that the function $f_{1}$ is a probability density for T₁ once the values of the parameters obtained from the estimate of the mixture cure model, together with the exponential distribution parameter, have been fixed. In this regard, because in general this does not hold for whatever parameter value, we might see that the distribution of T₁ deviates significantly or very slightly from being a probability density. In the last case, we have that $T^{*}$ is approximately the sum of T₁ and T₂. This concept is of great importance because the density of T₁ is used to derive the $S_{1}$survival function, which is necessary to calculate the probability of interest, i.e. the probability of progression or recurrence to distant metastatic cancer.

To provide a full picture of the impact of these limitations on the results presented in our analysis, we show which combinations of covariates give us parameter values that do not allow $f_{1}$ to be exactly a density function. To prove whether $f_{1}$ is a valid probability density or not we verified the two conditions

$f_{1}(t)\geq0$ (a)

$\int_{0}^{\infty} f_{1}\left( t \right) dt =1$ (b)

In the present analysis (i.e., using real world data) the condition (a) is not necessarily always satisfied. The combinations of parameters for which f_1_(t) < −10^4^ are reported in the **Supplementary Methodological** **Table 1** below, in which we have set the following equations.

$f^{*}\left( t \right)=\frac{k}{\lambda^{k}}t^{k-1} e^{- \left( \frac{t}{\lambda} \right)^{k}}$ (c)

$f_{2}\left( t \right)= ϴe^{-ϴt}$ (d)

$f_{1}\left( t \right)=f^{*}\left( t \right)+\frac{1}{ϴ} \frac{d}{dt}f^{*}\left( t \right) .$ (e)

However, we can see that in those cases the distribution $f_{1}$ deviates so slightly from being a probability density that this can justify using this distribution to calculate the risk of developing a distant metastatic disease. Weibull and Log-Logistic distribution for the times of those not cured were used in our analysis, but we could not have considered an exponential distribution, for example. The reason is that this probability density is never equal to zero when it is evaluated at *t*=0, but because we have assumed $T^{*}$ to be a convolution of two positive variables, we have that the density of $T^{*}$must be zero at *t*=0, since this corresponds to an integration between zero and zero for the definition of convolution, as in equation (f). Therefore, additionally, it’s important to choose only probability densities for $T^{*}$ that allows taking the value zero in zero at least for some sets of reasonable parameter values.

$f^{*}\left( t \right)=\int_{0}^{t} f_{1}\left( t_{1} \right)f_{2}\left( t-t_{1} \right)dt_{1} .$ (f)

**Supplementary Methodological Table 1: Value of the parameters of**  $\boldsymbol{f}_{\boldsymbol{1}}$  **distribution (f) for which the conditions (a) is not satisfied. Italy. 1997-2017.**

|  |  |  | **Parameters of** $\boldsymbol{f}_{\boldsymbol{1}}$ **distribution** | | |  |  |
| --- | --- | --- | --- | --- | --- | --- | --- |
| **Stage** | **Age** | **Period of diagnosis** | **λ** | ***k*** | **ϴ** | **value of *t* corresponding to the minimum of *f_1_**** | **Value of *_1_*** |
|  |  |  |  |  |  |  |  |
| III | 15-44 | 1997-2017 | 7.87 | 1.44 | 0.26 | 24.97 | -0.000125 |
| III | 15-44 | 2007-2017 | 7.87 | 1.44 | 0.23 | 21.02 | -0.000520 |
| III | 45-54 | 2007-2017 | 7.87 | 1.44 | 0.26 | 24.23 | -0.000163 |
| I-III | 15-44 | 2007-2017 | 10.36 | 1.56 | 0.23 | 30.35 | -0.000123 |
|  |  |  |  |  |  |  |  |

*The function was tested for *t* values between 0 and 100 years since diagnosis.
